# Beef cows with larger vulvar width have greater antral follicle count, viable oocytes, and higher circulating AMH

**DOI:** 10.1590/1984-3143-AR2024-0077

**Published:** 2025-02-24

**Authors:** Renata Maculan, Gisvani Lopez de Vasconcelos, Jesús Alfonso Sánchez Viafara, Gabriel Miranda Moreira, Cintia Vanin, Nathalia Alves, Marcos Brandão Dias Ferreira, José Camisão de Souza

**Affiliations:** 1 Instituto Federal do Sul de Minas, Machado, MG, Brasil; 2 Departamento de Zootecnia, Universidade Federal de Lavras, Lavras, MG, Brasil; 3 Facultad de Ciencias Agrícolas y Veterinarias, Universidad de Santander, Valledupar, Colombia; 4 Grupo Investigación y Desarrollo en Sistemas Agropecuarios, Unidad de Investigación Ganadera, Centro de Desarrollo Tecnológico del Cesar, Valledupar, Cesar, Colombia; 5 Departamento de Medicina Veterinária, Universidade Federal de Lavras, Lavras, MG, Brasil; 6 Empresa de Pesquisa Agropecuária de Minas Gerais, Belo Horizonte, MG, Brasil

**Keywords:** reproductive efficiency, external genitalia, hormonal marker, fertility, viable oocytes

## Abstract

Owing to the low heritability of reproductive traits, the search for markers and their interrelationship that could indicate reproductively superior individuals is important in the selection process for bovine reproductive efficiency. This study aimed to investigate the possible interrelationships between the antral follicle count (AFC), vulvar-width (VW), anti-Müllerian hormone (AMH) concentrations, fertility in *Bos Taurus* and *Bos Indicus* females. Brahman (*Bos Taurus-Indicus*, *n =* 126) and Simmental and Angus (*Bos Taurus-Taurus*, *n =* 155) cows were classified as having large (≥86 mm) and small (<86 mm) VW. From each group, one blood sample per animal was collected to determine the AMH serum concentrations. The GLIMMIX procedure in SAS^®^ was used to determine whether vulva width (VW) and AMH classes, associated or not with breed, could influence the age at first calving (FCA), calving to first service interval (CFSI), calving interval (CI), number of services per pregnancy (SP), and number of viable oocytes (VO). Antral follicle count (AFC) (36.10 ± 1.90 vs. 22.78 ± 1.64, for large and small VW, respectively), AMH (1.17 ± 0.07 vs. 0.48 ± 0.007 ng/mL), and viable oocytes or VO (18.86 ± 1.76 vs. 10.15 ± 1.49) were greater (*P* < 0.05) in the large VW than in the small VW. Brahman cows had greater AFC (36.30 ± 1.34 vs. 22.09 ± 1.67), VW (106.94 ± 15.83 vs. 69.78 ± 14.11 mm), and AMH (1.18 ± 0.07 vs. 0.42 ± 0.05 ng/mL) compared to that of taurine cows. In conclusion, VW was an efficient predictor of AFC and AMH concentrations in both genetic groups, but under the conditions of this trial no link could be detected between these variables and the reproductive indices studied.

## Introduction

The selection of bovine females from reproductive indices is limited because of the long generation interval and low heritability of the traits (Silva et al., 2005). Bovine female fertility is measured using reproductive indices such as age at first calving (FCA) and calving interval (CI) (Perotto et al., 2006). However, such indices are subject to the action of non-genetic factors, such as nutrition and management practices (Zink et al., 2011). Therefore, the search for traits that can indirectly indicate reproductively superior animals is essential.

The reproductive structure sizes can affect fertility in bovine females. Ovarian size is a good predictor of follicular reserve and ovarian function (Modina et al., 2014). The external genitalia (vulva) can predict ovarian follicular reserve and fertility measurements in the field, such as the CI and reproductive efficiency (Mesquita et al., 2016; Maculan et al., 2018). Therefore, it is necessary to investigate the factors that can affect the size of the external genitalia and the possible differences between the genetic groups.

The ovarian follicular reserve is estimated by the antral follicle count (AFC) using ultrasonography. Antral follicle count has a positive association with the results from the use of reproductive biotechnologies (Santos et al., 2016). However, the possible relationships between AFC and reproduction indices remain controversial. Thus, additional studies are necessary to assess the associations of fertility indicator traits (AFC, vulvar measurements, and ovarian volume) with reproductive performance.

Anti-Müllerian hormone (AMH) acts as a modulator of follicular atresia and is an indicator of a greater ovarian follicular reserve (Monniaux et al., 2013).

This study aimed to assess the interrelationships between antral follicle count (AFC), vulvar-width (VW), anti-Müllerian hormone (AMH) concentrations and reproductive efficiency indices in *Bos Taurus* and *Bos Indicus* females.

## Methods

All procedures and protocols were approved by the Ethics Committee on the Use of Animals (Permanent Commissions/PRP-UFLA) of the Universidade Federal de Lavras, Lavras, Minas Gerais, Brazil (protocol number 063/15).

### Animals and facilities

The experiment was conducted on the Casa Branca Agropastoril farm (a commercial enterprise) in Careaçu, MG, Brazil. *Bos Indicus* (Brahman breed, *n =* 124) and *Bos Taurus* (Simmental and Angus breeds, *n =* 155) were used to identify differences in the genetic group regarding AFC, vulvar width (VW), and AMH concentration. The mean age of zebu females was 4.8 ± 2.7 years (2–9 years) and that of taurine females was 5.7 ± 3.2 years (2–16 years). Zebu cows weighed 317–774 kg (mean of 531.00 ± 98.62 kg), while the taurine cows weighed 258–803 kg (mean of 533 ± 95.72 kg). All cows were on *Brachiaria* sp. Pastures throughout the trial, supplemented with corn silage, *ad libitum* mineral mix, and concentrate at 1.5% of body weight. Reproductive management consisted of natural service and conventional artificial insemination (AI) or fixed-time artificial insemination (FTAI); all cows were cycling and body condition scores (BCSs) ranging from 3 to 8 (1–9, lean to obese; Thomas and Bailey, 2021). The BCS was recorded by a single evaluator. Cows with signs of estrus, those at the end of gestation (30 d to calving), and those in the recent postpartum period (less than 20 d after calving) were excluded from the study. Animals were classified by parity into two groups: N (nulliparous or primiparous, *n =* 117) and V (multiparous, *n =* 164).

### AFC and ovarian size

To determine the AFC, the population of antral follicles ≥3 mm in diameter from both ovaries was assessed using transrectal ultrasonography (Aloka SSD 500; Mure, Japan) with a B-mode linear transducer (5.0 MHz) on a random day of the estrous cycle. *Bos Indicus* cows were classified as high (≥50 follicles, *n =* 37), intermediate (30–49 follicles, *n =* 70), or low AFC (<30 follicles, *n =* 76) (Rodrigues et al., 2015). *Bos Taurus* cows were classified as having high AFC (≥25 follicles, *n =* 39), intermediate AFC (16–24 follicles, *n =* 16), or low AFC (≤15 follicles, *n =* 44) (Ireland et al., 2007; Burns et al., 2005). Ovarian size (OS) was measured by ultrasonography. All cows were evaluated by the same professional. Ovaries were classified (OSC) according to their longitudinal axes as S (small, ≤2.5 cm), M (medium, 2.5–3.5 cm), and L (large, ≥3.5 cm).

### Morphometry of VW

**Figure 1:**
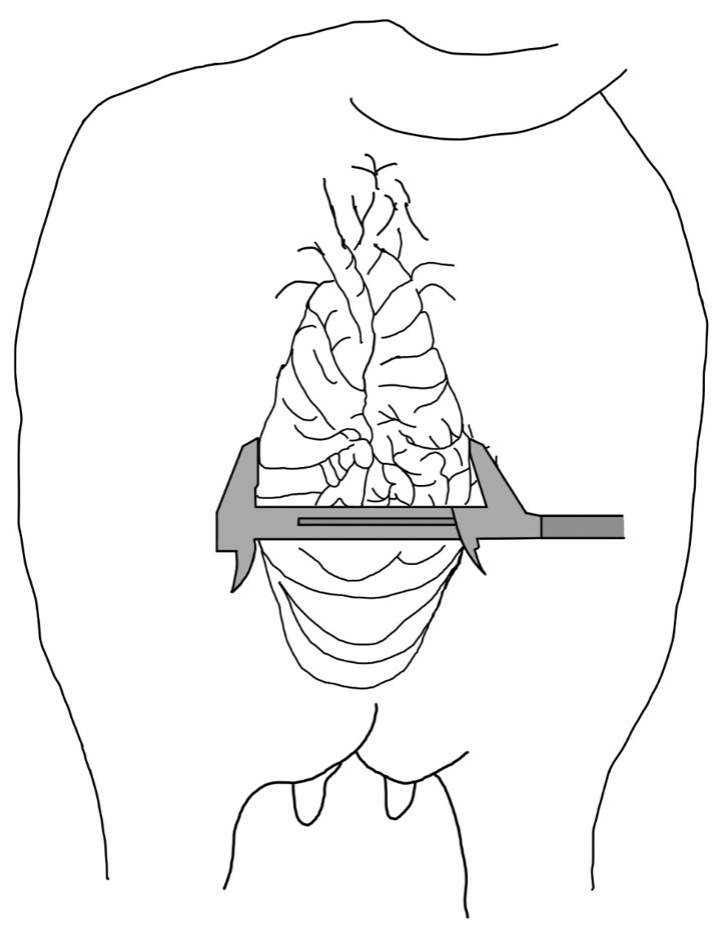
Vulva width measured with a digital caliper.

The morphometry of the external genitalia was measured using a digital caliper (150 mm/0.01 mm Powerfix Nf, Digimess, São Paulo, BR). Vulvar width (VW) was defined as the distance between the side edges of the vulva and the middle point of the rima length (opening) at an angle of 90° (Figure 1) (Mesquita et al., 2016), based on the frequency distributions of the values obtained for VW (L ≥ 86 mm and S < 86 mm).

### Evaluation of reproductive efficiency

Reproductive efficiency was evaluated from the reproductive history of females, from the farm records. The variables analyzed were age at first calving (FCA), calving to first service interval (CFSI), calving interval (CI), number of services per pregnancy (SP), and number of viable oocytes (VO) in cows subjected to follicular aspiration protocols.

### Blood sampling and determination of AMH

Blood samples were collected by coccygeal venipuncture in 10 mL vacuum tubes (BD VACUTAINER®, São Paulo, BR) on a random day of the estrous cycle. Serum was obtained by centrifugation at 3000 × g for 15 min and were frozen and stored at -20°C for later analyses. Serum AMH concentrations were determined by enzyme-linked immunosorbent assay kit (AnshLabs, Webster, Texas, USA), as previously validated for bovines (Ireland et al., 2008). Three assays were performed with sensitivity of 0.011 ng/mL with intra-assay and inter-assay variabilities from 1.8 to 2.8, and 0.4 to 0.8 respectively. From the respective frequency distributions, the animals were classified as high (>0.81 ng/mL) and low (≤0.81 ng/mL) AMH. The assays were conducted in the IgAc (Instituto Genese de Análises Científicas, São Paulo, BR) laboratory.

### Statistical analysis

All statistical analyses were performed using SAS^®^ (Cary, NC, USA, 2016). Classes of VW and the AMH concentrations were created based on the respective frequency distributions. General data were tested for normality using the Shapiro–Wilk test according to the UNIVARIATE procedure and, when necessary, transformed to achieve normality. The GLIMMIX procedure was used to assess AFC, FCA, CFSI, CI, SP, and mean number of VO according to BCS, parity, VW class, AMH class, and interactions. Data are presented as mean ± standard error of the mean. Differences were considered statistically significant by the Tukey test at *P* < 0.05.

## Results

### AFC, reproductive track morphometry, AMH and reproduction

Antral follicle count (AFC) was higher (*P* < 0.0001) in zebu (36.30 ± 1.34) than in taurine females (22.09 ± 1.67). Variation in the total count ranged from 4 to 119 in zebu females and 1 to 88 in taurine. VW was greater in cows in the high AFC class. AFC was similar between age (*P* = 0.19) and parity (*P* = 0.14), regardless of the breed. Ovarian size did not differ between the breeds; however, AFC was higher in the large ovarian class (Table 1).

**Table 1:** Ovarian size class (OSC)*, antral follicle count (AFC) and vulvar width (mm).

**OVARY**	**OSC**			***P***
	**SMALL**	**MEDIUM**	**LARGE**	
	**(<2.5 cm)**	**(2.5-3.5 cm)**	**(>3.5 cm)**	
	**AFC**			
**Right**	17.08 ± 13.29^c^	29.30 ± 18.60^b^	41.69 ± 27.93^a^	< 0.05
	(*n =* 81)	(*n =* 132)	(*n =* 62)	
**Left**	20.20 ± 14.15^c^	31.16 ± 20.49^b^	52.34 ± 32.74^a^	< 0.05
	(*n =* 121)	(*n =* 126)	(*n =* 26)	
	VULVAR WIDTH (mm)**			
	SMALL	MEDIUM	LARGE	
**Right**	78.12 ± 2.56^c^ (*n =* 82)	88.51 ± 2.02^b^ (*n =* 132)	92.70 ± 2.90^a^ (*n =* 64)	<0.001
**Left**	82.02 ± 2.12^c^ (*n =* 121)	87.41 ± 2.08^b^ (*n =* 126)	99.16 ± 4.41^a^ (*n =* 28)	<0.001

* Ovaries were classified (OSC) according to their longitudinal axes as S (small, ≤2.5 cm), M (medium, 2.5–3.5 cm), and L (large, ≥3.5 cm). ** Vulvar width was classified and analyzed over for the right ovary and left ovary separately

In the same row, superscript letters indicate significant differences (*P* < 0.05). The numbers are the mean values of the least squares ± standard error of the mean.

The VO number was greater (*P* < 0.05) in the cows of the large (18.86 ± 1.76, *n* = 38) than in those of the small VW class (10.15 ± 1.49, *n* = 53).

Reproductive indices (FCA, CFSI, CI, and SP) were similar between AFC classes (*P* > 0.05).

In zebu, VW was greater (106.94 ± 15.83 mm; *P* < 0.0001) than in taurine cows (69.78 ± 14.11 mm). The VW ranged from 35.15 to 99.24 mm in taurine females and from 67.67 to 143.37 mm in zebu females. Vulvar with (VW) which was smaller (P<0.05) in heifers (81.01 ± 2.17 mm, *n =* 115) than in cows (90.11 ± 1.81 mm, *n* = 165), was greater in larger ovaries (Table 1).

Antral follicle count (AFC) was greater (P < 0.0001) in the large VW class (36.10 ± 1.90 mm) than that of the small class (22.78 ± 1.64 mm). This result was still observed when analyses considered the genetic groups (Table 2).

**Table 2:** Vulvar width class (VWC), antral follicle count (AFC) and anti-Müllerian hormone (AMH) concentrations (ng/mL) according to the genetic group

	**VWC**				
	**LARGE**		**SMALL**		***P***
	***n***	**AFC**	***n***	**AFC**	
**Taurine**	11	29.27 ± 5.18^a^	144	21.53 ± 1.46^b^	<0.0001
**Zebu**	108	36.80 ± 2.30^a^	17	33.12 ± 5.80^b^	<0.0001
	***n***	**AMH**	***n***	**AMH**	
**Taurine**	11	0.39 ± 0.02^a^	144	0.43 ± 0.05^a^	> 0.05
**Zebu**	108	1.23 ± 0.09^a^	17	0.89 ± 0.02^b^	< 0.05

Superscript letters in the same row, superscript letters indicate significant differences (*P* < 0.05). The numbers are the mean values of the least squares ± standard error of the mean.

The AMH concentration in the large VW class (*n* = 103, 1.17 ± 0.07 ng/mL) was superior (*P* < 0.0001) to that of the small VW class (*n* = 119, 0.49 ± 0.07 ng/mL). However, there was an interaction between the genetic groups, such that this result was not maintained in taurine females (Table 2).

The AMH concentration in zebu cows was greater (*P* < 0.0001) than that in the taurine females (1.18 ± 0.07 ng/mL, *n* = 112, and 0.42 ± 0.05 ng/mL, *n* = 110, respectively).

The AMH concentrations did not decrease with age, regardless of the genetic group (*P* > 0.05), and were similar between parities, regardless of the genetic group (*P* = 0.84). AMH concentrations did not differ between BCS in either genetic group (*P* > 0.05). AMH concentrations in both genetic groups were higher in the large ovary class (Table 3).

**Table 3:** Ovarian size class (OSC) and anti-Müllerian hormone (AMH) concentrations (ng/mL) according to the genetic group

	**GENETIC GROUP**							
	**ZEBU**				**TAURINE**			
**OSC** *	**RO**	**n**	**LO**	**n**	**RO**	**n**	**LO**	**n**
**Small**	1.02 ± 0.02^c^	31	1.06 ± 0.01^b^	50	0.29 ± 0.01^c^	51	0.36 ± 0.07^b^	71
**Medium**	1.25 ± 0.02^b^	63	1.04 ± 0.01^b^	58	0.36 ± 0.09^b^	69	0.53 ± 0.07^a^	68
**Large**	1.45 ± 0.03^a^	31	2.17 ± 0.02^a^	15	0.68 ± 0.01^a^	33	0.43 ± 0.02^b^	13

* Ovarian class in which each ovary (left or right) was included independently of the cow, yielded distinct AMH values. RO: right ovary; LO: left ovary. Superscript letters in the same column indicate significant differences (*P* < 0.05). The numbers are the mean values of the least squares ± standard error of the mean. Small (<2.5 cm), medium (2.5-3.5 cm), large (>3.5 cm).

There was no difference in reproductive indices between AMH concentration classes (*P* > 0.05).

The AFC in the zebu cows classified as high AMH was greater (34.54 ± 1.84) than that observed in low AMH zebu cows (23.08 ± 1.74) (*P* < 0.0001). This difference was not observed in taurine cows (Table 4).

**Table 4:** High Anti-Müllerian hormone (AMH) class are related to higher antral follicle counts (AFC) in zebu, but not in taurine females.

	**ZEBU**			**TAURINE**		
**AMH CLASS** **1**	**n**	**AFC**	***P***	**n**	**AFC**	***P***
**High AMH**	78	40.81 ± 2.60^a^	0.003	52	25.13 ± 2.39^a^	>0.05
**Low AMH**	46	28.65 ± 3.40^b^		99	20.49 ± 1.73^a^	

^1^High AMH- >0.81 ng/mL; Low AMH- ≤0.81 ng/mL. Superscript letters in the same column indicate significant differences (*P* < 0.05). The numbers are the mean values of the least squares ± standard error of the mean.

## Discussion

Vulvar width in zebu and taurine females was associated with AFC, corroborating previous studies (Mesquita et al., 2016; Maculan et al., 2018). The development of the reproductive tract is influenced by genes (e.g AMH, WNT-A, GATA-4, DHH) which in turn, exert effects on ovarian function including follicular development (Richards, 2001; MacLaughlin et al., 2001). This was evidenced in correlation observed between the ovarian reserve and the development of reproductive tract and external genitalia (Cushman et al., 2013; Honaramooz et al., 2004; Jimenez-Krassel et al., 2009; Maculan et al., 2018; Mesquita et al., 2016).

However, it is not certain whether these positive correlations are due to the action of common genes which stimulate both, folliculogenesis and the development of the reproductive tract or whether it corresponds to specific factors produced by the follicles that exert influence on the reproductive tract development (Cushman et al., 2024).

Additionally, we must consider that, during the reproductive life of the cow, these genes may undergo epigenetic alterations caused by environmental or external factors. Thus, it is possible that cows of similar chronologic age may actually have distinct epigenetic ages. Among these alterations there are distinct ratios between histone deacetylases and methyl transferases abundances, beside DNA methylation (Clarke et al., 2021; Zhu et al., 2023; Klutstein and Gonen, 2023).

Thus, there are cows with characteristics that indicate fertility and others that suggest subfertility due to alterations in the epigenome, causing effects on the follicular cumulus oocyte complex and on the development of the reproductive tract in opposite directions, altering the phenotype match (Cushman et al., 2023; Mossa and Evans, 2023).

Vulvar width (VW) was greater in cows with higher AMH concentrations and AFCs. A direct explanation for this finding it is not clear, however a recently reported AMH effect may help to elucidate it, which is related to the regulation of the uterine function (Ferdousy et al., 2020). This finding strengthens the already known AMH effects on the quality of bovine oocyte (Vasconcelos et al., 2020; Torres-Rovira et al., 2014; Nguyen et al., 2022) and on the regulation of the ovarian function (Cushman et al., 2002; Gigli et al., 2005; Estienne et al., 2015; Yang et al., 2017; Juengel et al., 2021). Bovine oviducts and uteri have a Type 2-AMH receptor of which no reports on abundance changes throughout the estrous cycle or reproductive senescence have been published yet (Ferdousy et al., 2020). This allows to infer that decreases on AMH circulating concentrations may influence fertility by the regulation of uterine function. Subfertility is related to the depletion of the number of follicles by reproductive aging (Ferdousy et al., 2020).

Lower uterine weight, ovarian weight and endometrial diameter were observed in subfertile ewes and cows (Cushman et al., 2023). This suggests that the size of the reproductive tracts is correlated with fertility since subfertile ewes had lower AMH concentrations.

Moreover, infertile ewes have lower AFC and AMH, and smaller reproductive tracts. However, there are some discrepancies, where there are ewes with ovarian volumes which are in contrast with the fertile/infertile classification, e.g. ewes with larger ovaries and low fertility, and vice versa. Nevertheless, fertile ewes always have superior AMH concentrations (Cushman et al., 2023). In subfertile cows, this correspondence was also observed (Cushman et al., 2013; McNeel et al., 2017; Snider et al., 2022), and lower AFCs were observed in subfertile ewe (Torres-Rovira et al., 2014). This is further supported by the regulation of uterine function by AMH (Ferdousy et al., 2020).

The VW of zebu females was greater than that of taurine females. As mentioned before, AMH is related to fertility and to the development of the reproductive tract (Cushman et al., 2023; Ferdousy et al., 2020), facilitating the understanding of why taurine cows have smaller VW. The overall AMH concentration in zebu cows was higher than in taurine cows. The concentration of AMH is greater in females with intermediate and high AFC. Zebu cows have higher AFC than taurine (Maculan et al., 2018). This may be explained by the greater AMH production occurring in the pre-antral and early antral follicles (Durlinger et al., 2002a). Bos Indicus cows have less primordial follicles compared to Bos Taurus, but the AFC is higher in the former (Silva-Santos et al., 2011). It is believed that AMH participates in the inhibition of the primordial follicle activation mechanism, which triggers its ensuing growth (Durlinger et al., 2002b; Fortune et al., 2011). Thus, it is possible to understand the higher AMH concentrations in Indicus compared to Taurine cows observed in the current report.

The AMH concentration exhibited an association with the number of viable oocytes (VO); however, no association was observed with the reproduction indices in the field and cows classified as having high AMH had higher AFC and with the VO number. It is well established that AMH inhibits the production and follicular sensitivity to follicle-stimulating hormone, thus reducing follicular atresia (Ireland et al., 2009). In other words, AFC increases owing to greater circulating AMH concentrations even in zebu females that do not have a larger number of preantral follicles compared to taurine animals (Silva Santos et al., 2011; Batista et al., 2014). As mentioned before, it may be suggested that a lower atresia rate may lead to a greater number of viable oocytes.

In the present study, ovarian volume was associated with AFC. Ovarian size (OS) may predict the ovarian reserve considering that the hormonal production in the estrous cycle may regulate follicular size (Kwee et al., 2007; Penitente-Filho et al., 2015). Additionally, the gestation period exerts regulatory roles in the OS, which may impact its function and morphology (Chacur et al., 2006).

Antral follicle count is related to various fertility measurements, and the results observed in this study in relation to reproduction are promising, where AFC was associated with a larger OS, VW was associated with high AFC class and in turn AFC was associated with large VW class in both genetic groups.

A direct relationship between ovarian reserve and fertility is still unclear. A number of studies relate higher progesterone concentrations to better oocyte quality and higher follicle counts (Jimenez-Krassel et al., 2009; Martinez et al., 2016; Santa Cruz et al., 2018). However, other reports observed the opposite, where a lower number of follicles linked to higher progesterone concentrations (Lima et al., 2020; Bonato et al., 2022). Moreover, it is important to consider that there is a correlation between the ovarian reserve and external factors, such as temperature, nutrition and health which induce epigenetic modifications in genes associated to fertility, helping to explain the divergent results related to variations in fertility and their relation with AFC (Cushman et al., 2024).

Antral follicle count was greater in zebu females classified as having high AMH. This association has been clarified in the literature (Rico et al., 2009; Ireland et al., 2010; Batista et al., 2014; Souza et al., 2016; Maculan et al., 2018); however, this relationship was not observed in taurine females in the present study. The AFC and AMH concentrations were highly variable, and the AMH concentration was greater in zebu females. Corroborating these findings, Batista et al. (2014) observed a larger number of follicles and higher AMH concentrations in *Bos Indicus* than in *Bos Taurus* females. Insulin-like growth factor I (IGF-1), in addition to the well-established AMH/AFC link, may help to understand this result, since it is related to higher oocyte quality. Insulin-like growth factor I (IGF-1) concentrations are higher in *Bos Indicus* compared to *Bos Taurus* cows, and this is associated to higher AFC in the former (Alvarez et al., 2000; Satrapa et al., 2013).

## Conclusion

In conclusion, this study demonstrates that VW is a good predictor of AFC and AMH concentrations in both the genetic groups. Ovarian size, vulvar width, and hormonal traits were positively associated with the number of viable oocytes, antral follicle count and AMH, and can be used in models directed toward the selection of *Bos Taurus Taurus* and *Bos Taurus Indicus* females for superiority in these traits.
